# Assessment of Anaesthesia Teams' Non-Technical Skills in Clinical Practice before and after Simulation-Based Team Training: A Quasiexperimental Study

**DOI:** 10.1155/2024/2021671

**Published:** 2024-07-05

**Authors:** Anne Strand Finstad, Conrad Arnfinn Bjørshol, Ingunn Aase, Jo Røislien, Randi Ballangrud

**Affiliations:** ^1^Department of Nurse Anaesthesia, Division of Emergencies and Critical Care, Oslo University Hospital, Oslo, Norway; ^2^SHARE-Centre for Resilience in Healthcare, Faculty of Health Sciences, University of Stavanger, Stavanger, Norway; ^3^The Regional Centre for Emergency Medical Research and Development (RAKOS), Stavanger University Hospital, Stavanger, Norway; ^4^Department of Anaesthesiology and Intensive Care, Stavanger University Hospital, Stavanger, Norway; ^5^Department of Clinical Medicine, University of Bergen, Bergen, Norway; ^6^Faculty of Health Sciences, University of Stavanger, Stavanger, Norway; ^7^Department of Health Science, Norwegian University of Science and Technology, Gjøvik, Norway

## Abstract

**Background:**

In situ simulation-based team training of non-technical skills is considered an important initiative for preventing adverse events caused by poor teamwork among healthcare personnel. This study aimed to assess the non-technical skills of anaesthesia personnel before and after in situ simulation-based team training in a clinical setting.

**Methods:**

The study was conducted from October 2020 to June 2021 using a quasiexperimental before and after design based on video-recorded observations and ratings of anaesthesia teams' non-technical skills during anaesthesia induction in the operating room before and shortly after in situ simulation-based team training. Anaesthesia personnel were divided into 20 teams and video recorded during anaesthesia induction. The Anaesthetists' Non-technical Skills (ANTS) system was used to score the teams' non-technical skills. A paired-sample *t*-test was used to assess the impact of the intervention on the anaesthesia teams' scores on the various ANTS categories. Interrater agreement between the two ANTS raters was assessed using weighted kappa.

**Results:**

At the category level, the overall scores had a statistically significant increase in performance after simulation-based team training (3.48 vs. 3.71; *p* < 0.001). Furthermore, scores of five of the 15 elements were significantly different. Interrater agreement revealed moderate agreement between the two raters (weighted kappa = 0.51, *p* value <0.001).

**Conclusion:**

The anaesthesia teams' increased non-technical skills after simulation-based team training may indicate the transfer of knowledge from training to clinical practice. The moderate agreement between the raters could be attributed to the subjective nature of the evaluation procedure. The ANTS was originally used as an individual assessment tool; however, this study has demonstrated its potential as a team assessment tool.

## 1. Background

In hospitals, teamwork can cause intraoperative errors, adverse patient outcomes, and mortality. Non-technical skills are important in prevention of adverse events [1, 2]. Non-technical skills are defined as “the cognitive, social, and personal resource skills that complement technical skills” [3]. The World Health Organization (WHO) patient safety action plan 2021–2030 deems the integration of patient safety competencies including non-technical skills, in simulation-based team training to be critical [4]. A meta-analysis [5] stated that there is substantial evidence for simulation-based education to improve non-technical skills. However, there is still a lack of research regarding transfer of learning to anaesthesia teams' clinical practice.

High-fidelity human patient simulation was introduced in healthcare by anaesthesiologist David Gaba [6], with anaesthesia personnel as pioneers in the implementation of human factor-based simulation-based team training [7, 8]. Among anaesthesia personnel, simulation-based team training including task management, teamwork, situation awareness, and decision making prepares teams to manage challenging situations and is considered essential for safe clinical practice [9, 10].

In team training involving anaesthesia personnel, improved team performance [11, 12], cultural attitudes, and communication [13] after simulation-based team training of non-technical skills have been reported. A recent study by Finstad et al. exploring anaesthesia personnel's experience from interprofessional in situ simulation-based team training of non-technical skills reported that training contributed to enhanced anaesthesia practice [14]. The study focused on transfer of skills learnt from simulation-based team training to clinical practice with respect to level one (trainee reactions), level two (trainee learning), and level three (transfer of learning to clinical practice) of Kirkpatrick's four-level model [14, 15]. As the research so far has been mainly focused on Kirkpatrick levels one (reaction) and two (learning), further research on levels three (behaviour) and four (results) is needed [16]. A systematic review concluded that further research on the retention and transfer of human factors including non-technical skills from simulation-based team training to clinical practice is essential to characterize its effects on patient safety [17].

Boet et al. concluded in their systematic review, with presentation of assessment tools to assess teamwork, that there is a need for tools to assess intraoperative team performance and that selection of tools depend on the specific context [18].

Fletcher et al. highlighted the importance of both technical and non-technical skills in all circumstances that arise during different healthcare procedures. In addition, they introduced the Anaesthetists' Non-technical Skills (ANTS) system as a framework for both observing and rating non-technical skills for development of overall good practices in anaesthesia [19, 20]. The ANTS system is a tool designed for experienced anaesthetists to rate non-technical skills of another anaesthetist with at least basic technical competence [21].  Table 1 provides an overview and description of the ANTS system categories. The framework was introduced in aviation for assessing non-technical skills of pilots [22], and adaption to the health sciences led to behavioural rating systems for several professionals including anaesthetists [19], nurse anaesthetists [23, 24], scrub practitioners [25], and surgeons [26]. Even though the ANTS is used extensively [27–30], it is limited to skills that can be identified through observable behaviour.

Despite widespread implementation of simulation-based team training in anaesthesia, we do not know to what extent non-technical skills in anaesthesia teams is learned during this training. Assessment and feedback using the ANTS system can be used in both clinical and simulation environments to identify strengths and weakness [19]. The system is used to evaluate performance and is an indicator of patient safety [31]. Moreover, it is used when anaesthesia is normally administered in the operating room.

Assessment of non-technical skills during anaesthesia induction in clinical practice before and after simulation-based team training could help in evaluation of the transfer of learning from simulation to clinical practice [14, 17]. Therefore, this study aimed to assess the non-technical skills of anaesthesia personnel before and after in situ simulation-based team training in a clinical setting.

## 2. Methods

### 2.1. Design

The study used a quasiexperimental before and after design [32] using video recordings of the anaesthesia teams' non-technical skills during anaesthesia induction in the operating room. All videos showed two anaesthesia personnel working with other members of the surgical team. Overall, 20 videos before and 20 videos after simulation-based team training were viewed and evaluated by experienced nurse anaesthetists.

### 2.2. Setting and Sample

The impact of simulation-based team training was assessed during anaesthesia induction with endotracheal intubation in patients undergoing ear-nose-throat surgery. A total of seven anaesthesiologists and nine nurse anaesthetists (total *n* = 16) employed in a surgical department of a university hospital in Norway were recruited for the study. Nearly all participants had substantial work experience but varying experience with simulation training. The participants were paired into 20 different teams consisting of either an anaesthesiologist and a nurse anaesthetist (*n* = 17) or two nurse anaesthetists (*n* = 3). The same teams were assessed before and after simulation-based team training.

### 2.3. In Situ Simulation-Based Team Training Programme

The in situ simulation-based team training of non-technical skills was developed based on categories in the ANTS system (Table 1) to define learning objectives and scenario (Table 2). The simulation-based team training programme was structured based on the simulation setting model by Peter Dieckmann [33, 34] (Table 3).

The in situ simulation-based team training programme was conducted in March and April 2021. The anaesthesia team (one anaesthesiologist and one nurse anaesthetist) on duty participated in the simulation-based team training with the simulated case of anaesthesia induction. The focus was only on the anaesthesia team, but two operating room nurses participated as support during the anaesthesia induction simulation to create realism. Before the simulation-based team training, the participants received an information leaflet regarding healthcare simulations and non-technical skills. Each simulation-based team training session lasted for one hour. Simulation-based team training scenarios were led by an educated facilitator (ASF) in an operating room. The facilitator directed the debriefing and concluded with a summary and evaluation.

### 2.4. Data Collection

**Figure 1:**
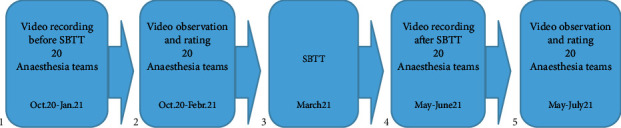
The study procedure.

Video recordings of the anaesthesia team in clinical practice in the operating room were conducted before and after in situ simulation-based team training. Overall, 20 videos were recorded before the simulation-based team training and 20 videos were recorded after the simulation-based team training (box 1 and 4, Figure 1). The video recordings were evaluated (box 2 and 5, Figure 1) by two experienced nurse anaesthetists with respect to non-technical skills based on the framework for observing and rating tool, ANTS system [20]. Figure 1 shows an overview of the study procedure.

#### 2.4.1. Instrument

In this study, the non-technical skills of anaesthesia personnel with respect to teamwork were assessed using the ANTS System Handbook [20]. The ANTS system comprises a level hierarchy (Table 4), with the highest level being the four categories task management, team working, simulation awareness, and decision making. These four categories of ANTS further consist of 15 skill elements. Each element is defined in the user manual, along with examples of good and poor behaviours (Table 5) and serve as behavioural markers, helping to indicate the presence or absence of the skill elements. Elements rate non-technical skills on a 4-point Likert-type scale (1 = poor, 2 = marginal, 3 = acceptable, and 4 = good) with an opportunity to respond and N=not observed. ANTS was translated into Norwegian using back translation [35] and used with permission (Rhona Flin, University of Aberdeen, 2019).

#### 2.4.2. Procedure

Two experienced nurse anaesthetists familiar with the language and structure of the ANTS system participated as raters [20]. In the preparation phase, the two raters underwent training in rating organised and conducted by the first author (ASF) to become familiar with the instrument and to increase the non-technical skills rating agreement between themselves. Video files from a pilot test in clinical practice and video recordings from anaesthesia students' simulation settings [36] were used for rater training. After watching the training videos, each of the two raters individually rated each of the two team members—the nurse anaesthetist and the anaesthesiologist—and then jointly re-evaluated their ratings to develop a common understanding of the rating procedure.

After the training, the two raters independently viewed each of the 20 before and 20 after videos of the anaesthesia team during anaesthesia induction, rating each team member individually according to the non-technical skills using the ANTS system framework. Team scores were calculated as the mean of the two individual team members' scores, as the ANTS system was not developed for assessing an entire anaesthesia team but only the anaesthesiologist, one of the two team members.

### 2.5. Data Analyses

For each of the 16 participants in the study, the scores for each of the 15 skill elements were calculated as the mean of the two raters, while the team score was calculated as the mean of the two team members. When an element for one team member was not observed, the observed team member's score represented the team score. One non-technical skills' score for one team was missing.

The analyses did not include the performance of the present operating room nurses.

The overall ANTS scores were summarised as the mean (SD) across all teams. The scores before and after simulation-based team training were compared using paired-sample *t*-tests. Statistical significance was set at *p* < 0.05.

ANTS scores for each item are ordinal variables, and interrater reliability was thus assessed using weighted kappa [37]. The following description of values and strength of agreement was used: <0.01 = none, 0.01–0.20 = poor, 0.21–0.40 = fair, 0.41–0.60 = moderate, 0.61–0.80 = good, and 0.81–1.00 = very good [38]. Data were analysed using IBM SPSS Statistics 28 [39].

The study adheres to the Transparent Reporting of Evaluations with Nonrandomized Designs (TRENDs) guidelines [40].

## 3. Results

### 3.1. Anaesthetists' Non-Technical Skills' Scores

**Figure 2:**
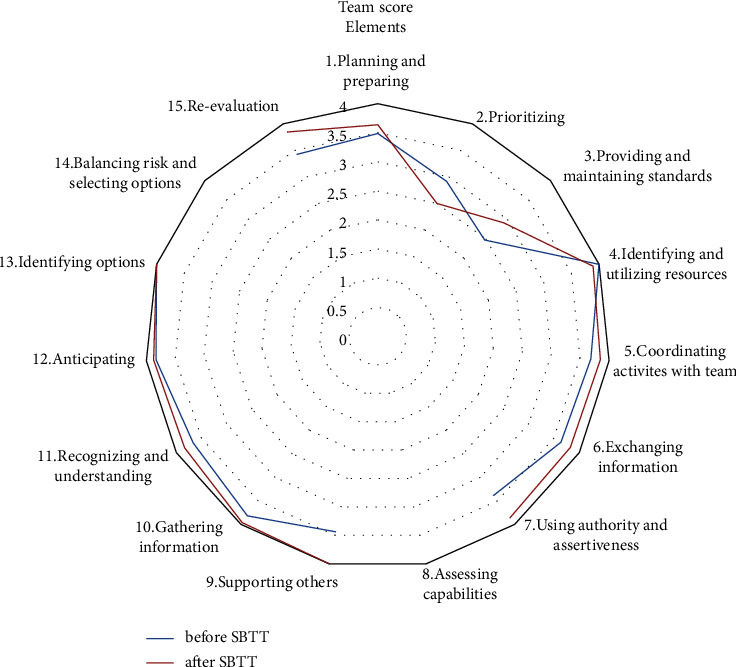
Radar diagram of ANTS team scores for each element before and after simulation-based team training.

The overall mean (SD) ANTS team scores before and after simulation-based team training were 3.48 (0.56) and 3.71 (0.45), respectively (*p*=<0.001), with a corresponding mean (95% CI) increase in ANTS scores of 0.23 (0.16–0.30) (Table 6). At the category level, the mean (SD) ANTS team scores had a statistically significant increase after the simulation-based team training intervention in three of the four categories. At the element level, the mean (SD) team ANTS scores had a statistically significant increase in five out of 15 elements (Table 7). While not statistically significant, the scores increased slight increases also in five other elements after simulation-based team training (Table 7 and Figure 2).

### 3.2. Interrater Reliability

Assessment of the agreement between the two raters yielded a weighted kappa value of 0.51, indicating moderate agreement.

## 4. Discussion

The overall ANTS team score had a statistically significant increase after simulation-based team training as did three of the four ANTS categories and five out of 15 ANTS elements.

### 4.1. Task Management

In the task management category, only the element “providing and maintaining standards” had a statistically significant increase in score after simulation-based team training, which may indicate that this element was more observable than the elements with minor increase or even decrease in performance. Kirkpatrick and Kirkpatrick claimed that observation assessment depends on the noticeability and measurability of the behaviour markers [15]. “Providing and maintaining standards” concerns safety and quality with measures including following protocols, cross-checking medication, checking machines, and maintaining anaesthesia journal. These behaviour markers may provide insights into elements of “planning and preparing” including preparing drugs and equipment. The elements are connected as they may have certain overlap with each other [20]. Moreover, the element “planning and preparing” was easier to rate than the element “providing and maintaining standards” in Jepsen et al.'s study conducted in a simulation setting [41]. This may explain the possible difference between measurement in simulation and clinical practice. Nevertheless, our results reveal a minor increase in task management, indicating transfer of learning from simulation to clinical practice.

### 4.2. Team Working

The teamwork category had a statistically significant improvement in scores after simulation-based team training with significant improvement in the elements “exchanging information” and “using authority and assertiveness.” These elements are crucial for team coordination and task completion (e.g., updating, confirming understanding, and maintaining documentation) and team leading and clear communication (e.g., state cases and providing justification) [20]. This improvement may confirm that teamwork is an important category for simulation-based team training for transfer of learning to clinical practice [42]. Even though the elements “co-ordinating activities with team members” and “supporting others” only had non-significant increase in performance, these elements may contribute to the improvement in the category with the other elements. No behavioural markers were observable in the element “assessing capabilities,” which may be due to the situation and behaviour observability [15]. Moreover, four of the five elements had team improvement, despite statistically significant increase in just two elements. However, we suggest that rater training should be prioritized beforehand, as the score system may seem simple during training but difficult in practice [41].

### 4.3. Situation Awareness

The team scores concerning situation awareness had statistically significant improvement at the category level and in the element “gathering information,” referring to collecting data about the situation by observing the environment including measures such as frequently scanning and cross-checking patient, surgery, and team information. This category is essential for patient safety [43] and to understand the observed aspects and predictions based on these observations [20]. The elements “recognizing and understanding” and “anticipating” had minor improvement in scores and contribute to the improvement in the scores of the category. This category's correlation to the increased teamwork scores is not surprising as these skills are likely overlapping. Focusing on these essential skills in the simulation-based team training may have contributed to improved performance in clinical practice.

### 4.4. Decision Making

This category concerns judgement for further action and the team scores had statistically significant increase in this category and the element “re-evaluating,” refereeing to continuous review of identified options, reassessment of different parameters including patient characteristics, situation, conditions evolved, and effectuated action [20]. No change was observed in the element “identifying options” with maximum scores before and after simulation-based team training. This element may be the strength of this category although it did not result in a statistically significant increase in performance. “Balancing risk and selecting options” was not observable most likely due to challenging observability [15] and/or may be the fact that some skills may take longer time to learn than was allowed in this study. Re-evaluation could be essential for all other categories concerning anaesthesia personnel's control of the situation, cooperation in the team, and task management for optimal patient care and safety.

### 4.5. In Situ Simulation-Based Team Training

A meta-analysis has shown that simulation-based education is favourable for improving non-technical skills [5]. Yet, simulation-based team training of non-technical skills for anaesthesia personnel with a view to transfer of learning to clinical practice seems to be lacking. Therefore, the results of our study might contribute to future simulation-based team training. In our study, the ANTS system categories [20] were used to define learning objectives for the in situ simulation-based team training program. To a certain extent, learning objectives were achieved with respect to calculated team score assessment in clinical practice after simulation-based team training. In situ simulation-based team training seems to provide the greatest opportunity to achieve improvement goals in team functioning [44]. Moreover, a single in situ simulation-based team training of the present learning objectives could be sufficient for certain goals. More frequent simulation-based team training could be needed to obtain even more transfer of learning to clinical practice, as recently reported [14]. The elements in ANTS may also be modified for specific clinical situations, which may have contributed to the statistical significant results in our study. This can be implemented for training but not for research, as the conditions can change suddenly [45].

### 4.6. ANTS System as a Team Score Instrument

All 15 ANTS elements were scored in this study. The “not observable” scores could be due to various factors including video-technical issues, difficulty of the raters to observe the real situation, or the subjective nature of the evaluation. The results depend on the noticeability and measurability of the behaviour [15], and a suitable assessment tool may be crucial. Abildgren et al.'s systematic review (2022) reports that non-technical skills including communication and teamwork, could be recognisable, and have valid interpretations for training personnel, but these elements may be difficult for the raters to assess. Rating non-technical skills is easier for passive observers, especially using prerecorded videos [17]. This observation agrees with the study by Ballangrud et al., describing self-report as important in assessing unobservable components [46].

The ANTS system has a high level of acceptability and a reasonable level of reliability [19]; in our view, this is a valuable assessment tool for anaesthesia teams in the operating rooms (ORs) even though the team scores were calculated as the mean of two individual scores [9, 18, 28].

Some studies suggest the need for development of robust team-based metrics [9, 18, 47, 48] and standards and recommend future research to determine if existing tools are suitable for OR teams [18, 47, 48]. The ANTS system has been suggested as basis for developing a team assessment tool in intensive care units [28]. This could also be considered for anaesthesia teams, as ANTS was originally developed for assessing individual anaesthetists. Boet et al. concluded that a number of team assessment tools are available for different teams but suggest more research into the topic of developing tools for intraoperative crisis situations, where anaesthesia teams are an integral part. Hence, we currently consider ANTS, and corresponding team scores calculated from individual assessments, to be a promising tool for team assessment of anaesthesia teams in clinical practice.

If the learning gained from simulation-based team training cannot be transferred to clinical practice, good reaction (level 1) or increased knowledge (level 2) in Kirkpatrick's evaluation model [15] are not relevant. Our results revealed increased performance in all the ANTS system categories and certain elements. Thus, the study revealed learning at level 3 in Kirkpatrick's model. Moreover, the study attempted to describe the change in clinical practice of experienced anaesthesia personnel following simulation-based team training. Their extended experience may have contributed to the moderate effect of simulation.

Although we used a team score calculated as the mean of two individual scores, further research is needed to develop a suitable team assessment tool based on the ANTS system.

### 4.7. Strengths and Limitations

The study had several strengths including the uniform team composition before and after simulation-based team training. Moreover, video recordings were used instead of raters being present in the OR, which helped to avoid disturbing the participants in the clinical setting. Furthermore, the raters could observe the video recordings multiple times.

However, our study has some limitations. The weighted kappa, a marker of rater agreement, showed only a moderate strength. Due to ethical approval and participants' consent in our study, the video recordings were deleted after three weeks, thereby limiting the raters available assessment' time. This could affect the rating owing to rater stress and fatigue [36]. The raters were aware whether they were rating before or after videos, which may be a weakness according to expectation of improved result after simulation-based team training. The raters decided their own training time. Therefore, the training period may vary from individual to individual and the readiness of each rater. Despite using a validated instrument, our participants were experienced clinicians which may have contributed to a ceiling effect with high score both before and after the simulation-based team training. This could also be the cause for the small variance in our data. ANTS is an individual assessment tool, and we calculated the team score from the mean scores of the two individuals. A team assessment tool suitable for the anaesthesia team would be preferable. However, since such a tool does not yet exist, the ANTS system was the best option available. Thus, good team performance could be achieved not only by one person performing well but also by the strength of the team itself.

## 5. Conclusion

The performance of anaesthesia teams improved in clinical practice after in situ simulation-based team training. This may indicate transfer of learning from simulation-based team training to clinical practice and aligns with level 3 (transfer of learning to clinical setting) in Kirkpatrick's evaluation model. The ANTS system categories may be helpful to prepare anaesthesia teams to cope with challenging situations and are essential for safe clinical practice. After simulation-based team training, the three categories, team working, situation awareness, and decision making, had statistical significant improvement due to the elements “providing and maintaining standards,” exchanging information”, “using authority and assertiveness,” gathering information” and “re-evaluating.” Observability may be low for certain elements that require more attention in future rater training; hence, there is a need to develop a more specific rating tool for team performance of NTS for intraoperative anaesthesia teams.

## Figures and Tables

**Table 1:** The ANTS system categories [20].

Task management:	Skills for organizing resources and required activities to achieve goals including individual case plans or longer-term scheduling issues

Team working:	Skills for working in a group, in any role, to ensure effective completion of collaborative tasks and team member satisfaction; the focus is particularly on the team rather than the task

Situation awareness:	Skills for developing and maintaining an overall awareness of the work setting based on observing all relevant aspects of the theatre environment (patient, team, time, displays, and equipment), understanding what they mean, and thinking ahead about what could happen next

Decision making:	Skills for selecting a course of action or diagnosis, in both normal conditions and in time-pressured crisis situations

Permission provided by the copyright holder.

**Table 2:** In situ simulation-based team training programme's learning objectives and simulation scenario.

Learning objectives	(i) The participants perform task management by displaying behaviour: planning and preparing, prioritizing, conducting standards, and using resources
	(ii) The participants perform teamwork by displaying behaviour: cooperating and communicating with team members, using authority and assertiveness, assessing capability, and supporting others
	(iii) The participants achieve situation awareness by displaying behaviour according to: gathering information, recognizing the environment, understanding what happens, and being anticipatory
	(iv) The participants perform decision making by displaying behaviour according to: identifying options, balancing risks and selecting options, and re-evaluating

Simulation-based team training scenario	Anaesthesia induction of elective patient for operation: septoplasty. The nurse anaesthetist prepares for anaesthesia induction: electronic documentation, monitoring equipment, anaesthesia machine and other required equipment, patient (simulated patient) preparations. The anaesthesiologist arrives in the operation room and coordinates with the nurse anaesthetist and patient. The anaesthesia induction starts (a manikin is used for intubation). The scenario ends when the endotracheal tube is inserted and its' position verified

**Table 3:** The present study's simulation-based team training programme phases [33].

1	The participants received an information leaflet regarding healthcare simulation and non-technical skills before the simulation-based team training, describing patient safety, simulation-based team training, non-technical skills categories and elements, and technical skills

2	The participants were briefed about the simulation environment, equipment, simulated patient safety, confidentiality, learning objectives (Table 2), and the scenario (Table 2)

3	The participants enacted a scenario (Table 2)

4	The participants attended a structured professional (and interprofessional) debriefing of the scenario actions, including reflections on what happened in this specific scenario, analysing good and bad actions, other possible solutions, and take home messages

5	The participants participated in an evaluation session, including discussions about what was learned

**Table 4:** The ANTS system categories and elements [20].

Categories	Elements
Task management	(i) Planning and preparing
	(ii) Prioritizing
	(iii) Providing and maintaining standards
	(iv) Identifying and utilizing resources

Team working	(i) Co-ordinating activities with team members
	(ii) Exchanging information
	(iii) Using authority and assertiveness
	(iv) Assessing capability
	(v) Supporting others

Situation awareness	(i) Gathering information
	(ii) Recognizing and understanding
	(iii) Anticipating

Decision making	(i) Identifying options
	(ii) Balancing risks and selecting options
	(iii) Re-evaluating

Permission provided by the copyright holder.

**Table 5:** Examples of ANTS elements with behaviour markers for good and poor practice [20].

Category	Element	Behavioural markers for good practice and poor practice
Task management	Prioritizing	Good practice: (i) Discusses priority issues in case (ii) Negotiates sequence of cases on list with surgeon (iii) Conveys order of actions in critical situations
		Poor practice: (i) Becomes distracted by teaching trainees (ii) Fails to allocate attention to critical areas (iii) Fails to adapt list to changing clinical conditions
Team working	Exchanging information	Good practice: (i) Gives situation updates/reports key events (ii) Confirms shared understanding (iii) Communicates case plans and other relevant information to appropriate people (iv) Maintains clear case documentation
		Poor practice: (i) Does not inform team of plan or subsequent alterations (ii) Gives inadequate handover briefing (iii) Does not include relevant people in communications (iv) Fails to express concerns in a clear and precise manner

Permission provided by the copyright holder.

**Table 6:** Anaesthetists' ANTS category scores for teams before and after simulation-based team training.

ANTS score categories	Before simulation-based team training *N* = 20 Mean (SD)	After simulation-based team training *N* = 20 Mean (SD)	Paired differences Mean (95% CI)	*p* value
Overall	3.48 (0.56)	3.71 (0.45)	0.23 (0.16–0.30)	<0.001
Task management	3.24 (0.34)	3.37 (0.25)	0.13 (0.04–0.30)	0.126
Team working	3.58 (0.25)	3.85 (0.12)	0.27 (0.14–0.39)	<0.001
Situation awareness	3.68 (0.27)	3.92 (0.16)	0.24 (0.11–0.36)	<0.001
Decision making	3.44 (0.45)	3.79 (0.37)	0.34 (0.10–0.58)	0.008

**Table 7:** Anaesthetists' ANTS element scores for teams before and after simulation-based team training.

ANTS categories	Element number	ANTS score elements	Before simulation-based team training *N* = 20 Mean (SD)	After simulation-based team training *N* = 20 Mean (SD)	Paired differences Mean (95% CI)	*p* value
Task management	1	Planning and preparing	3.49 (0.45)	3.64 (0.29)	0.15 (0.08–0.38)	0.186
	2	Prioritizing	2.92 (1.01)	2.50 (0.87)	0.42 (3.11–3.95)	0.662
	3	Providing and maintaining standards	2.48 (0.27)	2.91 (0.40)	0.44 (0.20–0.68)	0.001
	4	Identifying and utilizing resources	4.00 (0.00)	3.90 (0.28)	0.11 (0.16–0.37)	0.356

Team working	5	Co-ordinating activities with team members	3.69 (0.37)	3.85 (0.13)	0.16 (0.02–0.35)	0.079
	6	Exchanging information	3.64 (0.32)	3.83 (0.18)	0.19 (0.05–0.32)	0.010
	7	Using authority and assertiveness	3,38 (0.57)	3.87 (0.23)	0.48 (0.13–0.83)	0.010
	8	Assessing capabilities	…	…	…	…
	9	Supporting others	3.44 (0.52)	4.00 (0.00)	0.56 (0.26–1.38)	0.117

Situation awareness	10	Gathering information	3.81 (0.25)	3.96 (0.09)	0.15 (0.02–0.28)	0.024
	11	Recognizing and understanding	3.67 (0.52)	3.83 (0.41)	0.17 (0.26–0.60)	0.363
	12	Anticipating	3.83 (0.31)	3.88 (0.31)	0.04 (0.11–0.19)	0.551

Decision making	13	Identifying options	4.00 (0.00)	4.00 (0.00)	…	…
	14	Balancing risk and selecting options	…	…	…	…
	15	Re-evaluating	3.42 (0.43)	3.84 (0.34)	0.42 (0.16–0.68)	0.004

## Data Availability

The anonymized data scoring that support the findings of this study are available on request from the corresponding author. The video data are not available on request, when they are deleted due to privacy and ethical restrictions. No data are publicly available due to privacy or ethical restrictions.
